# Dengue Antibody Prevalence in German Travelers

**DOI:** 10.3201/eid1105.050097

**Published:** 2005-05

**Authors:** Ole Wichmann, Annekathrin Lauschke, Christina Frank, Pei-Yun Shu, Matthias Niedrig, Jyh-Hsiung Huang, Klaus Stark, Tomas Jelinek

**Affiliations:** * Berlin Institute of Tropical Medicine, Berlin, Germany;; † Robert Koch Institute, Berlin, Germany;; ‡ Center for Disease Control, Taipei, Taiwan, Republic of China

**Keywords:** Seroprevalence, dengue, travel, risk factors

## Abstract

We studied 2,259 German citizens after they returned from dengue-endemic countries from 1996 to 2004. Serotype-specific dengue antibodies indicated acute infections in 51 (4.7%) travelers with recent fever and 13 (1.1%) travelers with no recent fever, depending largely on destination and epidemic activity in the countries visited.

The number of dengue cases reported worldwide and the number of countries with endemic dengue activity has increased dramatically in recent decades (1). The disease is endemic in most tropical parts of the world, many of which are popular tourist destinations. Because of this popularity, and probably also because of heightened awareness, diagnoses of dengue in international travelers have increased as well (2–4).

Four dengue virus serotypes (DENV-1 to -4) are known to cause a wide range of clinical features (5); these viruses are transmitted mainly by *Aedes aegypti* mosquitoes. As in indigenous populations, a large proportion of asymptomatic infections are found in travelers (6,7). Recovery from an infection provides lifelong immunity against that serotype but confers only transient protection against heterologous infections (1), and sequential (secondary) infections may increase the risk of developing the more severe manifestation, dengue hemorrhagic fever (DHF) (8). DHF seems to occur rarely in European travelers, but several cases have been reported (9).

Previous studies demonstrated that the risk of acquiring dengue is highest when traveling in Southeast Asia (3,4,6). In that region, the disease is most prevalent during and after the rainy season, when vector breeding is maximal (10).

Only in a few industrialized countries is imported dengue fever a notifiable disease. In addition, substantial underdiagnosis persists because the disease is not well known to general practitioners in Western countries, and adequate diagnostic tests are available only in specialized clinics. Thus, little is known about dengue incidence in travelers over time. As dengue transmission may vary not only by season, but also from year to year, data derived from short-term observations may overestimate or underestimate the risk for travelers.

Determining the incidence of dengue in travelers is hampered by the fact that dengue immunoglobulin (Ig) G antibodies are broadly cross-reactive with other flaviviruses and vaccines against them (e.g., yellow fever and Japanese encephalitis). Previous studies to determine antibodies to dengue viruses by using commercially available enzyme-linked immunosorbent assay (ELISA) kits carry a risk for overestimation because of this cross-reactivity. Recently developed tests like the envelope/membrane (E/M) and nonstructural protein NS1 serotype-specific IgM ELISAs and NS1 serotype-specific IgG ELISA therefore offer new opportunities for a more reliable diagnosis and for determining the infecting serotype (11,12).

## The Study

To study the influence of increased worldwide dengue activity on international travelers, 2,259 patients were studied retrospectively for dengue antibodies after returning from dengue-endemic countries. A 36-month period from January 1996 to December 1998 was compared with a 27-month period from January 2002 to March 2004. We recruited travelers who came to the travel clinic of the Berlin Institute of Tropical Medicine, Germany, with fever (n = 1,091) or diarrhea without fever (n = 1,168) and for whom serum samples were available. Thus, 2,259 patients' serum samples were tested for anti-dengue IgM and IgG by using an IgM-capture ELISA and an IgG indirect ELISA (PanBio Pty Ltd., East Brisbane, Queensland, Australia). A probable acute infection was defined according to manufacturer's instruction as having a sample:calibrator absorbance ratio of IgM ≥1.0. Acute probable primary infection was characterized by the elevation of IgM ≥1.0 with IgG ≤4.0, and acute secondary infection was characterized by the elevation of IgG ≥4.0 (13). For a more specific diagnosis, all serum samples from patients with probable dengue infections were then investigated by using E/M and nonstructural protein NS1 serotype-specific IgM ELISAs and NS1 serotype-specific IgG ELISA as described previously (11,12). Furthermore, an additional confirmatory testing was performed by using immunofluorescence assays (Euroimmun AG, Luebeck, Germany), and if these results were contrary, sera collected during the acute phase of illness were processed by using polymerase chain reaction assays to detect viral nucleic acid.

Among the recruited patients, 1,163 (51.5%) were male. The median age was 33 years (range 2–79 years). Antibodies were detected by the screening test in 127 (5.6%) serum samples, indicating probable acute dengue infection. The more specific analysis confirmed infection in 64 cases (2.8% prevalence), including 8 (12.5%) patients with secondary immune response. One of these 8 secondary and none of the 56 primary infections led clinically to DHF. Among 1,091 patients with fever and 1,168 diarrhea patients without fever, 51 (4.7%) and 13 (1.1%), respectively, had an acute dengue infection.

The highest prevalence of dengue antibodies (4.6%), indicating acute infection, was found in patients returning from Asia (n = 1,020) (Table 1), including Southeast Asia (7.4% of 500 total travelers and 11% of 310 febrile travelers) and the Indian subcontinent (1.8%). Traveling in Southeast Asia was associated with a significantly higher risk compared to other disease-endemic areas in Africa and Latin America (odds ratio 5.3, 95% confidence interval 3.2–9.0). Comparing patients with and without acute dengue infection, no significant difference was seen in the median length of travel (28 vs. 24 days, respectively, p = 0.083, Mann-Whitney test) or the median age of the patients (32 vs. 33 years, respectively, p = 0.58, Mann-Whitney test). Patients 30–44 years of age had the highest antibody prevalence (37 [3.8%] of 966).

**Table 1:** Prevalence of anti-dengue antibodies in 2,255 patients according to travel destination and time*

Years	No. acute infections/total no. travelers (%)		
	Southeast Asia and Indian subcontinent	Sub-Saharan Africa	Latin America and the Caribbean
1996–1998	22/491 (4.5)	2/309 (0.6)	4/271 (1.5)
2002–2004	25/529 (4.7)	3/376 (0.8)	8/279 (2.9)
Total	47/1,020 (4.6)	5/685 (0.7)	12/550 (2.2)

*Four patients are not included in this analysis since they traveled to >1 continent.

When patients from 1996 to 1998 (n = 1,073) were compared with those from 2002 to 2004 (n = 1,186), a slight increase was seen in the overall prevalence, from 2.7% to 3.0%, although this finding was not significant (p = 0.63). The Figure shows annual dengue prevalence among travelers to Thailand and to the Indian subcontinent, highlighting that infection rates fluctuate strongly between years and between quarters within years. In the last quarter of 1997 and 1998, 64 travelers returned from Thailand, and 14 (22%) acquired an acute dengue infection. Among those, 5 were infected by the serotype DENV-1, 3 by DENV-2, and 4 by DENV-3 (Table 2). In 2 cases the serotype was undetermined.

**Figure:**
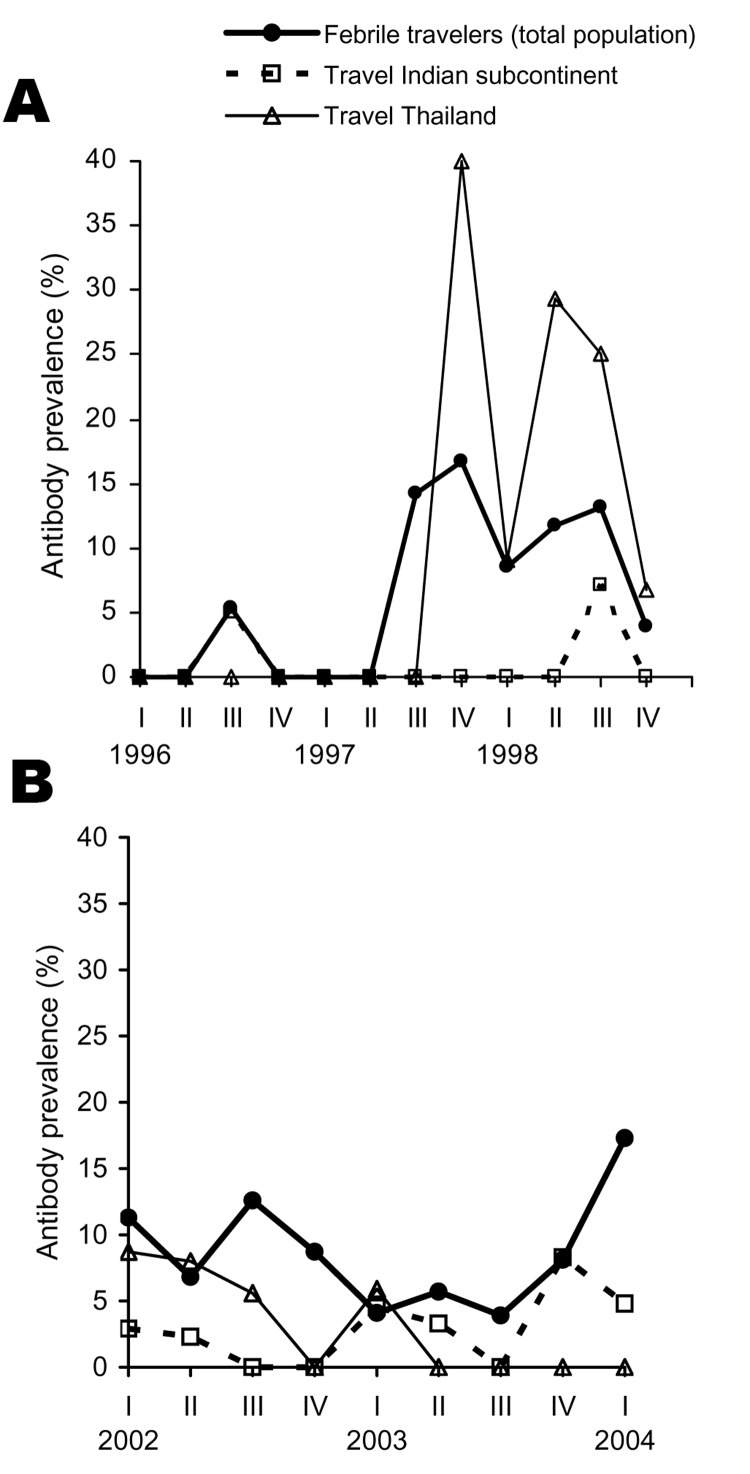
Prevalence of anti-dengue antibodies in travelers according to quarters of the year. Lines indicate acute dengue infection after returning from Thailand (n = 223) or from the Indian subcontinent (n = 495), both with and without fever, and in the total febrile population returning from all travel destinations (n = 1,091).

**Table 2:** Frequencies of dengue serotypes that caused infections in German travelers according to year of travel and country of origin

Country of origin	Dengue serotype (1996–1998)					Dengue serotype (2002–2004)				
	D-1	D-2	D-3	D-4	Unknown	D-1	D-2	D-3	D-4	Unknown
Thailand	5	3	4	–	2	4	1	2	1	–
Indonesia	–	–	2	–	–	3	1	3	–	–
Indian subcontinent	–	1	1	–	–	–	3	2	1	1
Rest of Asia	1	1	1	–	1	–	–	2	–	1
The Americas and the Caribbean	–	2	2	–		–	3	2	–	3
Africa	–	–	1*	–	1	2†	–	–	–	1

*Seychelles. †Angola and Kenya.

## Conclusions

In this study population, 4.7% of all febrile patients returning from different areas of the tropics had dengue antibodies that indicated acute infection. This number underlines the effect on international travelers to dengue-endemic areas. As long as no dengue vaccine is commercially available, the single-most effective preventive measure is avoiding mosquito bites. This advice should be included in every medical pretravel consultation. Among patients without fever, 13 (1.1%) had detectable dengue antibodies compatible with acute dengue infection, which underscores that symptoms commonly associated with dengue, such as fever, myalgia, arthralgia, and exanthema, are helpful for diagnosis when present, but the absence of typical symptoms does not exclude infection.

A study on 483 cases of imported dengue infections in Europe showed that DHF developed in 2.7% of the patients; immigrants from dengue-endemic countries returning to Europe after visiting their home country were at higher risk for more severe disease than Europeans (9). The immunologic status of those patients was unknown. However, immigrants from dengue-endemic countries have a higher prevalence of dengue antibodies from previous infections. In our population, all 54 patients with primary dengue infection had classical dengue fever, and 1 of 8 patients with secondary dengue infection had DHF. The patient with DHF was born in Sri Lanka and immigrated to Germany 2 decades ago. These observations might be taken as more evidence for the importance of letting patients know that they have been infected with dengue and should, therefore, protect themselves from infection with subsequent serotypes.

Fluctuations in prevalence between years, especially the maximal prevalence in 1998, correspond to similar observations on record. In a study conducted among Israeli travelers to Southeast Asia from 1994 to 1998, a sharp increase in incidence was noted in 1998 compared with previous years (14). Similar to the worldwide increase of cases reported to the World Health Organization, the number of Swedish travelers returning from Southeast Asia with dengue fever was considerably higher in 1998 than during previous years (3). Decreasing resources for vectorborne infectious disease prevention and control (15) might have contributed to this epidemic in Southeast Asia, which followed the economic crisis in 1997.

The risk among a cohort of Dutch short-term travelers to dengue-endemic areas in Asia from 1991 to 1992 showed marked seasonal variation for the Indian subcontinent (7). In our population, such seasonal variations were not detectable. The infection rates were more influenced by major outbreaks, such as the one in India in 2003 or in Southeast Asia in 1997 to 1998. Similar findings have been described for Israeli travelers during their trip to Thailand in 1998 (14) and in German travelers to Brazil and Thailand in 2001 and 2002, respectively (4).

To screen our samples, ELISA-based tests for IgM and IgG were combined. In a study performed with paired serum samples, combining IgG and IgM ELISAs had a sensitivity of 100% in primary infections and 99% in secondary infections. The specificity was 100% in non-flavivirus infections and 80% in Japanese encephalitis virus infections when an IgG sample:calibrator absorbance ratio of 3.0 was used as a cutoff (13). However, because our study was retrospective, only single serum samples were available from each traveler. To increase specificity, all serum samples that indicated probable infections were further investigated with more specific ELISA techniques. By using virus-infected culture supernatants as the source of viral antigens, the E/M-specific capture IgM has been found to differentiate reliably between Japanese encephalitis, dengue, West Nile virus, and yellow fever (12). Furthermore, an NS1 isotype- and serotype-specific ELISA can reliably differentiate Japanese encephalitis virus infection, Japanese encephalitis virus vaccination, and primary and secondary dengue virus infection (11). In primary infection, IgM is detectable 3–8 days from the onset of symptoms (8); thus, some of our travelers might have had false-negative test results if samples were taken during the acute phase of illness. Therefore, the true infection rates in our study might have been higher than the numbers indicated by single-sample serology.

Overall, we demonstrated an almost stable rate of dengue infections among Berlin Institute of Tropical Medicine patients returning from all tropical regions when recent years are compared with the mid-1990s. Large outbreaks like those in 1997–1998 in Southeast Asia (especially Thailand) and 2003 in major cities of India, all popular tourist destinations, contributed to the numbers. Quarterly and annual fluctuations might lead to misinterpretation of probable trends if data are derived only from short-term observations. In addition, this variability underscores the importance of tourists' seeking information before traveling to dengue-endemic areas.
